# 20 Years of Research on Socioeconomic Inequality and Children's—Unintentional Injuries Understanding the Cause-Specific Evidence at Hand

**DOI:** 10.1155/2010/819687

**Published:** 2010-07-25

**Authors:** Lucie Laflamme, Marie Hasselberg, Stephanie Burrows

**Affiliations:** ^1^Division of Global Health, Department of Public Health Sciences, Karolinska Institutet, Nobels väg 9, 171 76 Stockholm, Sweden; ^2^Centre de Recherche du Centre hospitalier de l'Université de Montréal, 1301 Rue Sherbrooke Est, Montréal, QC, Canada H2L 1M3

## Abstract

Injuries are one of the major causes of both death and social inequalities in health in children. This paper reviews and reflects on two decades of empirical studies (1990 to 2009) published in the peer-reviewed medical and public health literature on socioeconomic disparities as regards the five main causes of childhood unintentional injuries (i.e., traffic, drowning, poisoning, burns, falls). Studies have been conducted at both area and individual levels, the bulk of which deal with road traffic, burn, and fall injuries. As a whole and for each injury cause separately, their results support the notion that low socioeconomic status is greatly detrimental to child safety but not in all instances and settings. In light of variations between causes and, within causes, between settings and countries, it is emphasized that the prevention of inequities in child safety requires not only that proximal risk factors of injuries be tackled but also remote and fundamental ones inherent to poverty.

## 1. Introduction

Despite being regarded as highly preventable, injuries account for an increasing share of childhood mortality in the world [1, 2]. The most common causes of child mortality and morbidity by injury are road traffic crash, drowning, poisoning, burns, and falls. These injuries are unevenly distributed between countries [1, 3] and, within countries, between socioeconomic groups, to the detriment of the more disadvantaged families and communities. Reviews published throughout the years, be they focused on a specific childhood injury cause or setting [4–6] or covering several of them [7–11] strongly substantiate this notion. Injuries are in fact acknowledged as one of the causes of childhood mortality with the steepest socioeconomic gradient [1–15]. 

Despite this knowledge, in the medical and public health literature alone, a great deal of research is regularly published on socioeconomic disparities in childhood injury that specifically aims at measuring the magnitude of those differences. Some injury causes like traffic-related ones are frequently studied [2, 7–11], whereas others, like burns and drownings, receive far less attention. As this literature is highly descriptive in nature (as is the case for studies on socioeconomic differences in injuries in general), there is a paucity of studies on the mechanisms susceptible to generate those differences, which poses challenges to prevention work. Likewise, the reasons why differences exist in socioeconomic disparities across studies are seldom thoroughly addressed.

Some of the reviews mentioned above that, inspired by lead authors on social inequalities in health, put forward key mechanisms that help understanding why socioeconomic disparities may arise [4, 9–11] but they deal with the “why differences exist” and “how to combat differences” questions in generic terms and they provide little insight regarding why socioeconomic differences vary both in magnitude and direction, either with increasing age or across settings. This paper proposes to move this discussion forward. It considers the five leading causes of child mortality and morbidity mentioned above, thus allowing a more accurate coverage of the whole age spectrum [1] and helping to highlight whether and how socioeconomic disparities in injuries vary over age category and causes. 

As a basis for the paper, we revisited and updated two recent reviews commissioned by the World Health Organisation and conducted in sequence by our research team (see below). Here, we examine and reflect on two decades of studies (1990 to 2009) on socioeconomic disparities in injuries among children aged up to 18 years. The findings are discussed in light of various conceptual approaches to the understanding of the socioeconomic differences in health and safety. Implications for preventive efforts are also raised.

## 2. Review Methods

### 2.1. Literature Search and Update

The source reviews [10, 11] encompassed empirical studies on socioeconomic differences in injuries published in the medical and public health literature during the period 1990–2006 and, in preparing this paper, an additional three years were added (2007–2009). The articles initially sought for were original research articles that examined socioeconomic disparities in injury risk across socioeconomic groups—all ages and all injuries in the first review [10] and unintentional injuries in children in the second one [11]. The articles were obtained through a literature search in the databases of SafetyLit and the National Library of Medicine's Medline. For the former database, all studies included under “social disparities” were examined for relevance. For the latter database, English, French, Swedish, and Danish language studies published between January 1990 and December 2009 were identified using the keywords “injury or injuries or accident or accidents” in conjunction with “educational status or education or social class or socioeconomic status or occupation or income or social position or socioeconomic position or socioeconomic context or social context or deprivation or socioeconomic factors or socioeconomic characteristics or residence characteristics or neighbourhood” and “infant or infants or child or children or childhood or adolescent or adolescents or adolescence or youth”. Additional studies were also identified from the reference lists in selected articles and in those of the reviews listed above.

It is important to note that, in the injury field, the SafetyLit database has both breadth (number of journals included) and depth (coverage from each journal's backfiles). In fact, it has been found that of five commonly used databases, EMBASE, PsycINFO, PubMed/Medline, SafetyLit, and Web of Science (including the Science Citation Index and the Social Science Citation Index), the database with the greatest breadth and depth of coverage for journals that publish articles in the injury prevention and safety promotion (IPSP) field is SafetyLit [16]. The SafetyLit database coverage includes all IPSP-relevant journals from each of the listed databases and, for the journals that are found in the source lists of the other databases, a greater depth of coverage of backfile years. Further, the SafetyLit database contains articles from journals that are not included in any of the other listed databases.

### 2.2. Selection of Articles

Because of the wider scope of the original review, [10] and the change in focus in the subsequent updates (second review [11] and the current one), it is unfortunately not possible to specify the total number of articles “originally” identified from the literature searches that deal with socioeconomic differences in childhood injuries. As a consequence, we also cannot specify the number (or proportion) of them that meet the selection criteria presented below.

From the original literature searches, titles and abstracts were scanned for relevance independently by at least two of the authors. Full papers were then obtained to check for further relevance and procede with data extraction (see below). 

To be included in the current review, empirical studies were retained when: 1. they examined the relationship between socioeconomic status (SES) and injury at an individual- or area-level as the primary research question, 2. they considered one or several of the five major cause of mortality and morbidity among children, 3. they concerned children aged up to 19 years, and 4. they included denominators (i.e., population data rather than only injury data) and assessments of significance between groups or areas (e.g., testing for significance or providing confidence intervals). These latter criteria guaranteed a minimum level of strength for any single study and no further assessment of the quality of evidence was applied. The age upper limit was relaxed for traffic-related injuries as motor-vehicle driver (up to 24 years old).

Studies that were typically excluded are those considering “all injuries aggregated” (over 20 in a former review) [11] or “specific body parts” [10, 11] that lack both insight into the understanding of the phenomenon and useful information on which to design intervention strategies and influence policy.

A data extraction form was devised and used to record details from each study included. The details retained for the current paper can be found in the results Tables 2to 7. In case of disagreement between the reviewers, consensus decisions were reached.

## 3. Results

Table 1 presents an overview of the studies reviewed by injury cause (traffic being split into four categories), severity level (mortality versus morbidity), country, and type of relationship between SES and injury. The majority of the studies reviewed were conducted in high income countries and focused on nonfatal outcomes. As the bulk of those studies considered boys and girls simultaneously, this aspect will not be further reviewed in the remaining results.

### 3.1. Road Traffic Injuries

Road traffic injuries are by far the most studied cause of health disparities in the child injury field. The vast majority of these studies are from European countries (26 out of 37 studies), and from Sweden and the United Kingdom in particular. Both area- and individual-based studies are represented as well as two multilevel ones.

### 3.2. All Road Users Combined

Six studies investigated road traffic injuries combined for all kinds of road users. All but one study [17] showed a positive relationship between level of deprivation and road traffic injury [18–23]. A multilevel study from South Korea on young children up to 5 years showed that deprivation has a clear positive relationship with mortality by transport-related causes [19]. Another study from South Korea found that transport-related mortality among boys, 10–14 and 15–19 years, in families with low income were more than twice as high as the mortality among their peers in families with higher income [20]. For girls, however, they did not observe any differences [20]. This is partly supported by a study from Australia that showed an increased mortality inequality for motor vehicle accidents for boys, 0–14 and 15–24 years, but for females only in the age group 15–24 years [21]. Swedish studies, on the other hand, found a similar social patterning for both sexes [22, 23].

### 3.3. Pedestrians

Pedestrian injuries are the most studied type of transport-related injury. The majority of the studies are ecological and most of them examined nonfatal injuries. All studies show a positive relationship between individual socioeconomic disadvantage or deprivation of the living area and pedestrian injuries [24–45]. One of the studies observed that the association between deprivation and increased pedestrian casualties in England is stronger among children than among older age groups [31]. Children in the most deprived areas have up to a four times higher risk for pedestrian injuries than children in the least deprived. [31, 32] Additional studies from Canada and the US support this finding [27, 28, 30]. Similar findings were also reported in a study from Greece where less wealthy towns have twice as many pedestrian injuries compared to wealthier ones,[37] and in Sweden (Stockholm) where poor areas have approximately 90% higher risk than the most affluent areas for pedestrian injuries [46]. The study from Greece indicates that boys are disproportionately disadvantaged regarding pedestrian injuries when they reside in less wealthy towns [37].

### 3.4. Bicyclists

Individual-based studies from Sweden and the United Kingdom show that children in families with low socioeconomic position (measured in terms of parental social class, education or disposable income) are at greater risk for bicycle injuries [18, 24, 33, 34, 45]. These findings are in line with area-based studies from the United Kingdom, [26, 29, 32], Ireland [42] and Canada [27, 30] showing that children from the most deprived areas have significantly higher risk for bicycle injuries than their peers from less deprived areas. In contrast with this, Swedish area-based studies show that contextual socioeconomic attributes of the living area are not significant for injuries sustained as bicyclists [46, 47]. 

### 3.5. Motorcyclists and Moped Users

Motorcycle injuries were considered only in three studies, two from Sweden and one from Australia. The Swedish studies were based on individual data and showed a positive relationship between socioeconomic diadvantage and road traffic injuries as motorcyclists [24, 48]. These findings are supported by an area-based study from Australia showing that children in the most disadvantaged quintile were more likely to be hospitalized for motorcycle injuries than children in the least disadvantaged quintile [38]. Three Swedish studies focused separately on injuries among moped users. One individual-based study showed that children of unskilled workers have significantly higher odds for injuries as a moped user as compared to children of intermediate and high-level salaried employees [24] On the other hand, two area-based studies found that living in areas with higher levels of deprivation reduced the risk for moped injuries [39, 46].

### 3.6. Car Occupants

Area-based studies from Canada, Australia and the United Kingdom found that children from the most deprived areas have significantly higher risks for injuries as car occupants than their peers from the least deprived areas [29, 30, 38]. These results are in line with a multilevel study from Sweden that showed that, after adjusting for compositional factors, there was still unexplained area variability for injuries among motorvehicle riders [35]. Individual-based studies from Sweden and the United Kingdom showed that young people in the most disadvantaged families have an increased risk for injuries as a car driver compared to children in the most advantaged families [24, 34, 49, 50].

### 3.7. Other Unintentional Injuries

Tables 5 (individual level studies), 6 (area level studies) and 7 (multilevel studies) describe the studies that have dealt with causes of injuries other than road traffic-related ones. As the third column of each table indicates, most studies considered several causes at a time (sometimes including RTIs). In the text below, the cause specific results are presented.

### 3.8. Drowning

Drowning was considered in only three studies. A study from Bangladesh, based on an individual level household survey, found greater socioeconomic disadvantage was associated with greater drowning mortality and morbidity among the under five [51]. In South Korea, two national-level studies showed conflicting results [19, 20]. One, a multilevel study conducted among small children (0–5 years), found a positive relationship between area-level deprivation and risk of drowning, after adjustment for sex and individual level SES variables [19]. Considering older children (10–14 and 15–19 years) and parental income as an individual level indicator, the other Korean study found no evidence of mortality differences by income level for either sex or age group [20].

### 3.9. Poisoning

Of the eleven studies that examined socioeconomic disparities in poisoning injury, the majority are ecological (*n* = 7) and all but one (England and Wales) [18] examined nonfatal injuries. Except for one from Peru, [43] all were conducted in high-income (mostly European) countries. None compares different age groups.

Of the individual level studies, only the Peruvian study, among 0–18 year olds in Lima, [43] found no association between household poverty or parental education and child poisoning. The other three studies, from high-income countries, observed large socioeconomic disparities. A Danish national study on unintentional home injuries, adjusting for sex, age, distance from hospital and several family, and household factors, observed a gradient of increasing risk of nonfatal poisoning with decreasing parental education and income [52]. Similarly, Canadian children (<18 years) from low-income families had odds of poisoning injuries which were 60% higher than those children from well-off families [53]. Poisoning deaths were higher among children from low social classes in England and Wales [18]. 

Area-based studies generally found strong positive associations between socioeconomic disadvantage and poisoning. Studies on injury hospitalisations in children aged 0–14 years conducted in New South Wales (Australia) [38] and in Québec (Canada) [30] showed that children in the most deprived quintile had a 52% and 68% higher risk, respectively, than children in the least deprived quintile. In England, there was a clear gradient of increasing risk of poisoning with increasing deprivation among 0–4 years olds in the East Midlands [54] and among 0–14 years olds in Trent [32]. In the former study, it was also observed that gradients were particularly steep for benzodiazepines, antidepressants, cough and cold remedies, and organic solvents. Similarly, the two most deprived quintiles had significantly higher hospital admission rates for poisoning than the three least deprived quintiles in Wales [36]. 

In Stockholm, the results depended on the measure of SES. A higher concentration of people with low socioeconomic status—but not low material deprivation—was associated with poisoning among children aged 0–15 years [39]. In Ireland, although 0–12 year olds living the most deprived areas of north and west Belfast had 3.65 the risk of being treated in the emergency department for poisoning compared to those in the least deprived areas, the association failed to reach significance [42].

### 3.10. Burns

In the studies reviewed, the definition of what constitutes a burn differed, some studies only dealt with injuries related to fire and flames, other included scalds from hot liquids. In several studies a definition was lacking. Unlike other causes, an almost equal number of individual and area levels studies have been conducted. 

Three individual level studies were conducted in low and middle income settings and they were based on survey or questionnaire data gathered from caregivers. In the Ashanti region (Ghana), one study found that maternal education was not significantly associated with burn injuries (with evidence of a physical scar) among children aged 0–5 years [55]. The other two studies, both from Lima (Peru), found evidence of disparities. Among 0–17 year olds, low income and crowding were strongly associated with increased risk, and better maternal education had a protective effect [56]. In the other study, household poverty was associated with an increased odds of burn injuries but low education of the household head was not [43]. 

Individual level studies from high income countries showed strong positive associations between socioeconomic disadvantage and burn injuries. In England and Wales, deaths rates from burn injuries were 16 [18] and 38 [29] times higher among children from families with the least favourable occupational status compared to those from the most favourable ones. Burn incidence rates were also higher among Danish children from families with low income and low education [52]. When scalds by hot liquids and burns on cookers were analysed as separate categories of burn injuries, the socioeconomic differences increased. In Alberta (Canada), children (<18 years) from low-income families (defined as those receiving subsidies for healthcare insurance premiums) had considerably higher odds of burn injuries compared with children whose families required no financial assistance [53]. In contrast, income was not related to fatal fire events among children less than five in Tennessee, after adjustment for several maternal and child characteristics [57]. Although low maternal education was associated with a more than threefold increase in fatal fire events, confidence intervals were wide (see Table 5).

Area-based studies are predominantly from the United States and the United Kingdom. Low income of census tracts was associated with higher rates of burns (nonfatal and fatal cases combined) among 0–19 year olds in Dallas,[58] among 0–16 years olds in northern Manhattan,[28] and among 0–14 year olds in Philadelphia [59]. In Trent (1992–1997) socioeconomic gradients for burn and scald hospital admissions were marked, children living in the most deprived areas had a 3.5-fold higher risk than those in the least deprived areas [32]. A similar gradient was also observed in admission rates for 0–14 year olds in Wales [36]. 

Studies on hospitalisation for burns in children aged 0–14 years conducted in New South Wales [38] and in Quebec [30] also revealed strong positive associations between area deprivation and the risk of burn injury. And a study in Cape Town (South Africa) found that poor housing conditions, socioeconomic barriers, and child dependency were associated with children's (0–12 years) burns in a graded fashion [60].

Although a study in Stockholm found that a higher concentration of people with low SES increased the risk of burns/scalds among children aged 0–15 years, [39] moderate compared to low material deprivation was associated with reduced risk of burn injuries. The association between economic deprivation of the living area of children aged 0–12 years, and burns/scalds did not reach significance in north and west Belfast [42].

### 3.11. Falls

Almost all of the eighteen fall studies reviewed were area-based and examined nonfatal outcomes. Most were from Europe, from Sweden and the United Kingdom in particular. With the exception of some Swedish studies, [30, 40, 47, 61] socioeconomic disparities were examined for all ages combined.

Somewhat mixed results emerge from individual level studies. Low paternal social class was associated with an increased risk of fatal falls in England and Wales [41]. Similarly, low education and low income were independently associated with nonfatal falls among Danish children [52]. When examined as separate categories, the risk of falls from playground equipment increased with greater socioeconomic disadvantage but falls from bunk beds did not. A Swedish study showed that socioeconomic disparities were not constant across ages 0–19 [22]. Low parental SES, adjusted for parental country of birth, single parent home, and receipt of welfare benefits were associated with a slightly increased risk of falls only for the youngest group of children (0–4 years). In a Peruvian study, [43] fall injuries among 0–18 year olds were not associated with household poverty or with low parental education. However, the combination of household poverty *and *low parental education increased the odds of these injuries by 30%. 

Area-based studies similarly report mixed findings. This is particularly the case for studies that stratify by sex, age, or subdiagnoses of falls (e.g., falls from same level, falls from furniture). In Ireland, fall injuries were considerably higher in the most, as compared to the least deprived areas, but the difference in rates only reached significance for low falls (<1 metre) [44]. Results from three Swedish studies reveal considerable variations in socioeconomic disparities when considering various types of falls and looking at age group and sex of the child separately; [40, 46, 61] and even differences over time are reported [40]. Most strikingly, both aggravating and protective effects are reported. For instance, one of these studies, [61] among young children (0–5 years), reported protective effects (about 30%) for falls at the same level (the largest diagnosis) and from heights, and aggravating effects for falls from items of furniture (about 34%). No association was found with falls from playground equipment. Among older children (6–15 years), protective effects were found for falls from playground equipment, falls from trees and sports-related falls, while aggravating effects for material deprivation (moderate level only) were reported for falls on the same level. The study including a time perspective on falls among boys and girls in the age groups 10–14 and 15–19 years [40] observed that the association between area deprivation and fall injuries changed markedly over time among girls aged 15–19 years, from being protective in the early nineties (1993–95) to being aggravating in the early 2000s (2003–05). No such changes were observed among younger girls or among boys of both age groups, where associations were weak in both time periods. A recent Canadian study (Quebec) [30] also observed that associations between area material deprivation and hospitalization for falls among 0–14 year old children were only significant for particular types of falls. Deprivation was associated with an increased risk of falls from stairs and from a building, and with a decreased risk for falls on the same level. In contrast, an Australian study separated falls that occurred in the playground and those that did not, and found children in the most disadvantaged areas had a reduced risk of both kinds of injury compared with those in the least deprived areas [38].

Several other studies found a positive relationship between area socioeconomic deprivation and fall injuries. Two Canadian studies report significant differences across income quintiles. In Kingston, children in the poorest quintile had a 42% higher risk of fall injuries compared to children in the richest quintile [17]. In a study concerned with trends in socioeconomic disparities across urban areas of Canada, the risk of death from falls increased by 29% for each unit change in income quintile, from highest to lowest [44]. Similar results were observed in the United Kingdom and the United States. In Wales [36] and Trent [32], there were clear gradients of increasing hospital admission rates among 0–14 year olds with increased deprivation. Children from moderately and largely low-income census tracts in northern Manhattan had a 50% and 90% higher risk of severe fall injuries (including deaths) than those in better-off areas. 

Two area-based studies did not find a significant association. There was no association between area material deprivation or socioeconomic status and Swedish children's hospitalisation for fall-related injuries [39]. And in England, the association between an index of multiple deprivation and serious child fall injuries disappeared following adjustment for ethnicity, lone parent families, and households without a car [29]. In addition, a multilevel study in Korea found that district level deprivation, adjusted for sex and individual level SES variables, was not associated with fall mortality among 0–5 year olds [19].

## 4. Discussion

### 4.1. Characteristics of the Evidence at Hand

Children are vulnerable to injuries for various types of reasons [62] and, as they develop, their injury pattern changes [1, 2, 62]. By considering several injury causes, a broader spectrum of potential hazards is captured that better mirror this whole period of life. It is also possible to examine whether socioeconomic differences tend to remain constant across causes and settings. Unfortunately, not all injury causes have been studied for their socioeconomic patterning to the same extent and an “across-causes” examination is rendered more difficult. In particular, drowning has rarely been investigated, despite the importance of these injuries in child morbidity and mortality. An additional drawback is the strong bias of the knowledge at hand to the conditions prevailing in a few high income countries, the evidence therefore being mainly representative of some types of governments, economies, and forms of social stratification (see also below).

From a methodological point of view, despite 20 years of empirical contributions, studies remain predominantly descriptive, [4, 7, 9–11] and, for most injury causes, rather than being conducted at the individual level (using measures such as maternal or paternal occupation, education, class or income, and household economy), many studies are area-based (using measures such as neighbourhood deprivation, percentage of low income households, and percentage of unemployed). The choice of observation unit is also very much country-dependant, with many studies from the UK for instance being area-based while others from Sweden are individual-based. In the case of area-based studies, it can be argued that, while designs of that kind are regarded as much weaker than individual level ones,[4] contextual and environmental modification (reducing exposure by e.g., eliminating, modifying, or separating sources of danger) is regarded as a very powerful and effective measure for primary injury prevention [10]. An earlier review [6] and subsequent studies [19] also show that the living environment may play a role in childhood injury causation, independent of individual attributes.

An additional observation is the paucity of studies that present data for boys and girls separately or for different age strata. This is further discussed below.

### 4.2. Main Findings

Although differences in studies' characteristics (e.g., method, sample size, setting, and age range) and methodology (e.g., data collection tools, relationship characterization, and injury severity criteria) make comparisons complicated, a number of observations nonetheless arise. As expected from previous reviews, [4–11] there tend to be substantial socioeconomic disparities in injuries throughout childhood and in all major causes of unintentional injuries. Interestingly also, the magnitude of the differences varies between causes and, within causes, between contexts as well as, when considered, between age groups.

### 4.3. Why Is Lower Socioeconomic Status Associated with More Unintentional Injuries?

To explain the very existence of socioeconomic differences in injuries, the notion of “fundamental causes” is useful [63]. The fundamental causes theory suggests that a range of resources susceptible to protect one's health and safety are socially distributed, implying that people of higher SES hold an advantage in warding off threats to their—and their offspring's—wellbeing. The theory predicts that SES is more strongly associated with health outcomes for which prevention and treatment measures are known, which largely applies to unintentional injuries [1, 2]. Poverty and material deprivation are acknowledged risk factors of child injuries, [1, 2, 7–11, 62] with two broad mechanisms contributing to this. One obvious one is that children from poor families and from more deprived areas are more exposed to a wider range of hazards, [64] what Towner and colleagues call “proximate tier” [9] and others, differential exposure [10, 11]. An additional mechanism is that their care provider or themselves lack the means to protect themselves in their home or in their community (e.g., the means to afford safe equipments or devices), differential vulnerability [9–11]. In a recent review, Schwebel and Gaines have summarized the situation in the following manner: “(…) in homes where financial and temporal resources are limited, both tangible (e.g., smoke alarms, bicycle helments, and outlet covers) and intangible (e.g. parental supervision, parental teaching of rules about safety) mechanisms to prevent child injury are lacking or inadequate.” ([62, Pages 246-247]).

### 4.4. Why Are there Differences in Magnitude and Direction across Studies?

The theory of “fundamental causes” also predicts that the strength of the association between socioeconomic status and health would vary across countries (or settings) depending on how any given country (or setting) compensates for socioeconomic disparities. Compensation can occur in many different ways that are meaningful to combat socioeconomic inequality in health and safety. One is through social welfare policies that contribute to enhance either individual social mobility or individual living circumstances (e.g., housing and commuting conditions); thereby minimizing exposure in amount and in kind. One can also imagine that welfare policies render possible better equity in care, which in turn can reduce injury lethality and minimize differential consequences of those injuries sustained. 

Child pedestrian injuries for instance are associated with very steep social gradients in the UK, where only area-based studies have been conducted, and with negligible ones in Sweden, where studies are predominantly individual-based. It is unclear whether area-based differences would also apply at the individual level in the UK (i.e., the ecological fallacy coming into play). To explain the situation in Sweden, one can mention the existence of “safety-for-all” measures [65, 66] that have long been on the agenda of the Swedish transport sector (see a discussion in [10, 11]). But it is of note that equity-oriented measures from outside the transport sector are also very likely to have contributed to buffer the negative effect of lower SES, minimizing exposure disparities between socioeconomic groups. Measures with that potential are for instance employment policies (for both men and women) combined with child care services, child access to recreational environments (other than the street), and limited distance to and from school for all. The latter reduces both child exposure to traffic (in duration) and variability of exposure across living areas.

It is a considerable knowledge gap that so few studies were conducted in low/middle-income countries where the burden of injuries is far greater. The bulk of the evidence at hand stems from high-income countries and, very often, countries from northern Europe. This, in combination with the use of different indicators of SES between studies, impedes our ability to further explore how the current empirical evidence supports the theory of fundamental causes. 

Processes like globalisation, urbanisation, motorisation, and environmental change could negatively impact on child safety and increase differences both between and within countries [67]. Additionally, in times of economic difficulties, as is the case when working on this paper, some factors are likely to exacerbate socioeconomic disparities. Of note is disturbed parental supervision, [68] previously proposed as an explanation to increased infant mortality due to unintentional injuries in metropolitan California in time of economic recession. Also troubling is a reduction in individual insurance, such as that of insured motorists or house owners and tenants. During a recession, less basic societal investment in safety in the form of for example, built-in safety, routine maintenance and reparation, and non universal/restrictive access to trauma care will put additional responsibility on individuals who are already under pressure.

### 4.5. Age-Based Differences in Socioeconomic Disparities

Whereas the above helps clarifying differences between settings or countries, it does not bring much light on age-based differences in disparities within similar settings. It is possible that in an environment like the home, where small children spend a great share of their time, the vulnerabilities inherent to poverty are more detrimental to them (given their development process and their dependency on their caregivers) [2, 9]. As few studies have investigated the matter, it is difficult to draw conclusions. Nonetheless, studies indicate that, for an injury cause as common as falls, disparities are stonger in the younger children than in older ones—or disparities exist only in the young. 

Because poverty exacerbates home injury risks in the young, it is possible that home safety interventions based on the uptake of safe practices may not be fully adapted to the situation of children from lower SES. But there are examples of home safety education programs that have reached normative improvements—and some significant injury reductions—even in resource poor households and neighborhoods that can inspire policy and practive [69–71].

West for his part has suggested that a process of equalisation of socioeconomic differences in injury risks may arise at school ages and be more pronounced for a number of health outcomes, among which are unintentional injuries (accidents) [72]. West poses that during that period of life, factors other than family, home background, and neighbourhood (i.e., class-related factors) come into play and counteract the impact of class-related ones; a process of “class-patterning equalisation” occurs [72, 73]. Among other mechanisms, the school, peer group, and youth culture may strongly impact on children's life-style choices and behaviours) [74–76]. The process would run from childhood through to young adulthood but its impact is expected to be at a maximum during early youth (in secondary school). Still, according to West, in the case of unintentional injuries, equalisation would be effected by means of similarity in the activities pursued by young people from different social backgrounds in the school and peer-group contexts. To date, there has been very little support in the injury literature for this representation.

Should further studies of the like be conducted, gender differences would definitely need to be taken into account. It is indeed unfortunate that so few studies investigated whether socioeconomic disparities are more detrimental to boys than to girls or even, whether sex differences appear hand in hand with the increasing age of the child. Whereas we have known for a long time that boys have greater injury mortality and morbidity than girls, we do not know how the factors contributing to those differences are socially distributed—biology, cognition, socialization, and exposure opportunity [55].

### 4.6. Policy Implications—Preventing Injuries and Reducing Socioeconomic Disparities

Poorer chances of survival and poor health, when generated by social processes to the detriment of the less well-off, impede basic human rights [65, 66, 77]. Health inequities in child injury can be reduced—and avoided [64]. Sectoral examples of passive safety dealing with physical exposures show that tackling material deprivation in the home through better housing conditions, [64, 78] or modifying the traffic environment [64, 78–81] can do much to “level up” safety differentials between members of different social groups. Given that injuries are the leading cause of death and disability among children worldwide, abatement strategies of the like that reduce injury risks for all children can only be welcomed. 

One could posit that the more the injury cause varies across socioeconomic groups (the theory of fundamental causes applying), the less likely it is that behavioural interventions will make a significant contribution to either disparity or global risk reduction [10, 11, 71]. One eloquent example of the problem arising with behavioural or safe practice campaigns is the case of burn injuries and fires in the UK where socioeconomic disparities are huge and where interventions aiming at the installation of burn detectors in resources-poor areas, even if subsidised, all failed to achieve their injury reduction goals [82]. The same reasoning could apply to pedestrian injuries when the preventive measures put forward rely on enhanced parental supervision [10, 70]. 

Although numerous interventions have been evaluated and promoted as effective, few have been conducted that assess whether those interventions are equally effective in all socioeconomic groups (or areas) or if they help reduce differences between those groups. In other words, very few interventions have been evaluated for their potential in childhood injury inequality reduction.

Below is a summary of interventions discussed that have the potential to reduce both injury rates overall and socioeconomic disparities in injuries. Several of them have been discussed in earlier policy documents and reviews [1, 2, 9–11, 70, 71]

- *Safety-oriented legislation or regulation* that determines minimum standards and conditions under which a number of activities cannot be performed or imposes safe behaviours and practices that would not be largely adopted on a voluntary basis.
- *Level up the safety of the physical environment through “passive” safety *measures can be achieved through engineering and product development. It is a matter of “modifying”, “isolating”, “separating” or “eliminating” the sources of danger.
- *Community-based prevention programmes* that intend to tackle the safety level of communities by combining strategies like behavioural and environmental changes, in some instances together with enforcing legislation and subsidies.
- *Home safety education and home visit programmes *aiming at promoting safe practices in the home and also for the prevention of both unintentional and intentional injuries.
- *The creation of attractive places for recreation *as the fewer off-street play areas that are offered, the more the street environment becomes not only an area for traffic but also one for recreation.

## 5. Conclusions

The literature on socioeconomic disparities in child injuries is abundant but reviews are few. By reviewing the literature on several childhood injury causes, a broader spectrum of potential hazards is captured that better mirror this whole period of life. The findings at hand are biased to some causes (especially traffic-related injuries, burns and falls) and some high income countries. In the main, they offer support to the notion that low SES is often greatly detrimental to child safety. Injuries are highly preventable and socioeconomic differences in wealth need not be reflected in differences in safety. Variations between causes and, within causes, between settings and countries suggest that the prevention of inequities in child safety needs not only that direct mechanisms of injuries be tackled but also remote and fundamental ones inherent to poverty.

## Figures and Tables

**Table 1:** Overview of studies on socioeconomic disparities in childhood injuries by injury cause.

Injury cause		Severity outcome		Relationship between deprivation/ socioeconomic disadvantage and injury^a^		
Study analytical level	*N*	Mortality/morbidity (*n*)	Countries (*n*)	Positive	Negative	None
*Traffic aggregated (* *n* = 7)						
Individual-level	3	Mortality (2); Both combined (1)	South Korea (1); Sweden (1); United Kingdom (1)	3		
Area-level	3	Morbidity (2); Mortality (2)	Australia (1); Canada (1); United Kingdom (1)	2		1
Multilevel	1	Mortality (1)	South Korea (1)	1		
*Pedestrian (* *n* = 24)						
Individual- level	7	Morbidity (4); Mortality (3)	Peru (1); Sweden (3); United Kingdom (3)	7		
Area-level	16	Morbidity (13); Mortality (1); Both combined (2)	Australia (1); Canada (4); Greece (1); Ireland (1); Sweden (1); United Kingdom (7); USA (1)	16		
Multilevel	1	Morbidity (1)	Sweden (1)	1	1	
*Bicyclist (* *n* = 14)						
Individual-level	5	Morbidity (3); Mortality (2)	Sweden (3); United Kingdom (2)	5		
Area-level	8	Morbidity (8)	Australia (1); Canada (1); Ireland (1); Sweden (2); United Kingdom (3);	6	2	
Multilevel	1	Morbidity (1)	Sweden (1)		1	
*Motorcycle/moped (* *n* = 5)						
Individual-level	2	Morbidity (2)	Sweden (2)	2		
Area-level	3	Morbidity (3)	Australia (1); Sweden (2)	1	2	
Multilevel	0					
*Car occupant (* *n* = 17)						
Individual-level	7	Morbidity (6); Mortality (1)	Sweden (6); United Kingdom (1)	7		
Area-level	9	Morbidity (7); Mortality (1); Both combined (1)	Australia (1); Canada (2); Ireland (1); Sweden (2); United Kingdom (2); USA (1)	7	2	
Multilevel	1	Morbidity (1)	Sweden (1)	1		
*Drowning (* *n* = 3)						
Individual-level	2	Mortality (1); Both (1)	Bangladesh (1); South Korea (1)	1		1
Area-level	0					
Multilevel	1	Mortality (1)	South Korea (1)	1		
*Poisoning (* *n* = 11)						
Individual-level	4	Morbidity (3); Mortality (1)	Canada (1); Denmark (1); Peru (1); United Kingdom (1)	3		1
Area-level	7	Morbidity (7)	Australia (1); Canada (1); Ireland (1); Sweden (1); United Kingdom (3)	6		2
Multilevel	0					
*Burn (* *n* = 18)						
Individual-level	8	Morbidity (5); Mortality (3)	Canada (1); Denmark (1); Ghana (1); Peru (2); United Kingdom (2); United States (1)	7		3
Area-level	10	Morbidity (7); Mortality (1); Both combined (2)	Australia (1); Canada (1); Ireland (1); South Africa (1); Sweden (1); United Kingdom (2); United States (3)	8	1	2
Multilevel	0					
*Fall (* *n* = 18)						
Individual-level	4	Morbidity (2); Mortality (1); Both combined (1)	Denmark (1); Peru (1); Sweden (1); United Kingdom (1)	4		2
Area-level	13	Morbidity (11); Mortality (1); Both combined (1)	Australia (1); Canada (3); Ireland (1); Sweden (4); United Kingdom (3); United States (1)	10	4	7
Multilevel	1	Mortality (1)	South Korea (1)			1

Note ^a^As a study can have results for more than one analyses (e.g., for different SES measures or at two different time periods), the numbers here may exceed the number of studies.

**Table 2:** Individual-level studies for childhood road traffic injuries: summary of methodological features and results (*n* = 14).

Author & year country (city/region)	Outcomes	Age group data source^a^	SES measure	Analysis covariates	Results: the level of 95% is used for all confidence intervals (CI)
Cho et al. 2007 South Korea (whole country)	Death due to transport accident (all types) stratified by sex and age group	10–14 and 15–19 years R: death register, health insurance beneficiary dataset	Parental income (based on insurance contribution – 3 levels)	Cox proportional hazards model None	Boys with parents in the third income tertile have significantly higher mortality in transport accidents than those in the first tertile. Boys 10–14 (RR = 2.66; CI 1.8–3.9), 15–19 (RR = 2.15; CI 1.6–2.8). There were no significant differences for girls
Donroe et al. 2009 Peru (Lima)	RTI as pedestrian, severe enough to require medical consultation	0–18 years I: household survey with guardian or with child if aged 12 years and over	Poverty (2 levels, parental education (2 levels)	Logistic regression Sex, age, other SES, overcrowding, number of children in the home	Children in poor households have increased odds for pedestrian injury (OR=1.59; CI 1.2–2.2) compared to those in more affluent households. Children with parents with low level of education have increased odds for pedestrian injuries compared to children with parents with high education (RR = 1.91; CI 1.4–2.7)
Edwards et al. 2006 United Kingdom (England and Wales)	Deaths as pedestrian, car occupant, cyclist	0–15 years R: Population based death register	Family occupational status (8 levels)	Death rates (95% CI) None	Children from family with the least favourable occupational status had 20.6 (CI 10.6–39.9) times higher deaths as pedestrians, 5.5 (CI 3.1–9.6) times higher deaths as car occupants and 27.5 (CI 6.4–118.2) times higher for deaths as cyclists than children in the most advantaged families
Engström et al. 2002 Sweden (whole country)	Hospitalisations and deaths combined, RTI (all types) stratified by age	0–4, 5–9, 10–14, 15–19 years R: population and housing censuses, hospital discharge register, death register	Parental social class (4 levels)	Logistic regression, slope index of inequality, relative index of inequality Parents' country of birth, single parent home, receipt of welfare benefits	Children of unskilled workers have higher odds for traffic injuries than children with parents that are intermediate and high level employees: 5–9 years (adjusted RR = 1.36; CI 1.2–1.5), 10–14 years (adjusted RR = 1.23; CI 1.1–1.3), 15–19 years (adjusted RR = 1.52; CI 1.4–1.6)
Hasselberg & Laflamme 2005 Sweden (whole country)	Hospitalisations, RTI as car driver	16–23 years R: population and housing censuses, hospital discharge register	Household social class (4 levels) Parental education (3 levels)	Logistic regression None	Car drivers who were injured several times show a similar social distribution to that of drivers sustaining just one. However, drivers from self-employed households show greater odds of injury repletion compared to drivers with parents that are intermediate and high level salaried employees (OR=1.65; CI 1.0–2.7)
Hasselberg & Laflamme 2004 Sweden (whole country)	Hospitalisations, RTI as pedestrian, bicyclist and car passenger	1–14 years R: population and housing censuses, hospital discharge register	Household social class (7 levels) Household disposable income (quartiles) Parental education (3 levels)	Poisson regression, population attributable risk Child's age, mother's age at delivery	Low socioeconomic position of the household increases the risk of being injured in traffic as pedestrian (RR = 1.39; CI 1.2–1.7), bicyclist (RR = 1.34; CI 1.3–1.4) and car passenger (RR = 1.31; CI 1.1–1.6). This association is also shown for other measures of SEP such as low disposable income and low level of education. The highest population-attributable risks were related to family disposable income and were indicated for pedestrians and car passengers (19%–20%)
Hasselberg & Laflamme 2003 Sweden (whole country)	Hospitalisations, RTI as car driver	16–23 years R: population and housing censuses, hospital discharge register	Household social class (7 levels) Household disposable income (quartiles) Parental education (3 levels)	Poisson regression, population attributable risk Child's age, mother's age at delivery	The long-term effects of low parental social class (OR=1.62; CI 1.4–1.9) and low education (OR=1.76; CI 1.52–2.03) on RTIs are evident in the case of young drivers. Level of family disposable income is not related to RTI among young car drivers
Hasselberg et al. 2001 Sweden (whole country)	Hospitalisations, RTI as pedestrian, bicyclist, moped user, mc-user, car driver	2–24 years R: population and housing censuses, hospital discharge register	Household social class (7 levels)	Logistic regression, population attributable risk Child's age, mother's age at delivery	Children of unskilled workers have higher odds for injuries as pedestrians (OR=1.30; CI 1.1–1.5), bicyclists (OR=1.34; CI 1.3–1.4), moped users (OR=1.80; CI 1.6–2.0), motorcyclists (OR=1.80; CI 1.6–2.0) and car drivers (OR=1.75; CI 1.6–2.0)
Laflamme et al. 2004 Sweden Stockholm County	Hospitalisations and deaths combined, RTI as protected and unprotected road user	0–19 years R: population housing censuses, hospital discharge register	Household socioeconomic status	Relative index of inequality, Chi-squared test	Equalisation for older boys as bicycle users (13–15 years RII=1.64;0.9–3.0, 16–18 years RII=1.16; CI 0.5–2.7)
Laflamme & Engström 2002 Sweden (whole country)	Hospitalisations, RTI as pedestrian, bicyclist, motor vehicle passenger, motor vehicle driver	0–4, 5–9, 10–14, 15–19 years R: population housing censuses, hospital discharge register	Household socioeconomic status (4 levels)	Regression analysis Sex	Significantly higher odds for children (aged 5–9 and 15–19 years) of unskilled workers for pedestrian injuries than for those in higher socioeconomic groups (5–9 years RR = 2.33; CI 1.7–3.1, 15–19 years RR1.55; CI 1.2–2.0
Murray 1998 Sweden (whole country)	Police reported traffic accidents among young motor vehicle drivers	16–22 years R: national road administration database, population and housing census	Social class (8 levels), school achievement (based on school marks in the school-leaving certificate)	*T*-test, difference of proportions None	The school achievement and school attainment were lower among young people involved in injuries compared to a sample of young people not involved in RTIs (*P* < .001)
Roberts 1997 United Kingdom (England and Wales)	Death rates RTI (all types), cyclist and pedestrian in a collision with motor vehicle	0–15 years R: death register	Social class of father (6 levels)	Poisson regression None	Children in social class V are more likely to suffer traffic accidents compared to those in social class I (motor vehicle accidents, OR=1.11; CI 1.1–1.2, cyclists, OR=1.30; CI 1.2–1.4, pedestrian, OR=1.47; CI 1.4–1.5)
Roberts & Power 1996 United Kingdom (England and Wales)	Death rates for motor vehicle accidents and pedestrian accidents.	0–15 years R: population censuses, death register	Social class of the father (6 levels)	Poisson regression None	Children in disadvantaged families have more RTI in both periods (1979–83 and 1989–92) compared to children in more advantaged households (*P* = .001). The decline in mortality due to motor vehicle injuries and pedestrian injuries was smaller in the manual working class (23% decline; CI 16–28) than in the nonmanual working class (34% decline; CI 24–43)
Zambon & Hasselberg 2006 Sweden (whole country)	Police reported road traffic crash as a motorcycle driver	16–23 years R: population and housing census, hospital discharge register, national road administration database	Household social class (5 levels)	Logistic regression, population attributable risk	Low socioeconomic position increases the motorcycle injury risk of both minor (OR=1.66; CI 1.5–1.9) and severe (OR=1.64; CI 1.3–2.1) outcomes to an equal extent, without giving rise to a higher risk of severe outcomes

Note ^a^R=register; I=interview; Q=self-administered questionnaire.

**Table 3:** Area-level studies for childhood road traffic injuries: summary of methodological features and results (*n* = 21).

Author and year country (city/region)	Outcomes	Age group data source	SES measure	Analysis covariates	Results: the level of 95% is used for all confidence intervals (CI)
Adams et al. 2005 United Kingdom (Tyne, Northumberland, Wear)	Police reported RTI as pedestrian, cyclist, car occupantStratified by sex	0–16 years R: regional police register	Townsend deprivation index of enumeration districts (quintiles)	Logistic regression None	Deprivation increases the odds of RTIs as pedestrian 1998–2003 (boys RR = 2.7; CI 2.2–3.3 and girls RR = 2.6; CI 2.0–3.2), vehicle passengers 1998-2003 (boys RR = 1.2; CI 1.0–1.6 and girls RR = 1.1; CI 1.0–1.4). Decreasing differences between 1988 and 2003
Birken et al. 2006 Canada (urban areas	Deaths pedestrian collisions with a motor vehicle	0–14 years R: death register	Household income for census tracts (quintiles)	Poisson regression Age, sex	For each unit change in income quintile, from highest to lowest, the risk of death as pedestrian increased by 13% (CI 5%–22%)
Coupland et al. 2003 United Kingdom (Trent)	Hospitalisations, RTI as bicyclist, pedestrian or other transport injury	0–14 years R: hospital records	Townsend deprivation index for electoral wards (quintiles)	Poisson regression Rurality, percentage males, ethnicity, distance to nearest hospital	Children in deprived areas have increased risk for RTI compared to those in more affluent area in the years 1996 to 1997, but no significant change between 1992–1997 (pedestrian injuries RR = 4.0; CI 1.9–8.2 and bicycle injuries RR = 1.8; CI 1.2–2.6)
Dougherty et al. 1990 Canada (urban areas and Montréal)	RTI mortality and morbidity as pedestrian and bicyclist	0–14 years R: hospital records, police reported accidents	Median household income, rate of poverty among children under 18 years for census tracts (quintiles)	Relative rates with 95% CI None	The rate of RTI was four times higher for children living in deprived neighbourhoods compared to those in affluent areas (injury rate 168; CI 138–204 and 686; CI 622–756). Inequalities more pronounced for pedestrians than bicyclists. Socioeconomic inequalities in fatal injuries greater in girls than in boys.
Durkin et al. 1994 United States (Northern Manhattan)	RTI mortality and morbidity asmotor vehicle user, pedestrian	0–16 years R: injury surveillance system	Household income (3 levels), education (2 levels), unemployment (2 levels) for census tracts (quartiles)	Regression analysis, rate ratios with 95% CI None	The injury rate ratio for children in low-income neighbourhoods is higher than for children living in neighbourhoods with few low-income households (motor vehicle injuries RR = 2.5; CI 2.0–3.2 and pedestrian injuries RR = 3.1; CI 2.3–4.2)
Edwards et al. 2008 United Kingdo (England)	Hospitalisations, RTI as pedestrian, bicyclist, car occupant	0–15 yearsR: centralised inpatient register, population censuses	Index of Multiple Deprivation (deciles)	Negative binomial regressionEthnicity, % households with no car, % lone-parent families	Rates of serious injury were higher in the most deprived areas than in the least deprived for pedestrians (RR = 4.1; CI 2.8–6.0), bicyclists (RR = 2.6; CI 1.7–4.0) and car occupants (RR = 2.0; 95% 1.4–3.3)
Elmén & Sundh 1994 Sweden (Gothenburg)	RTI mortality (all types)	1–14 and 15–24 years R: cause of death register	Mean income for parishes (3 levels)	Mantel-Haenszel tests Calendar year	Successively increasing RTI mortality with lower socioeconomic status of the area for both men and women (*P* < .001)
Faelker et al. 2000 Canada (Ontario)	Traffic injuries seen in emergency departments	0–19 years R: Emergency department-based surveillance system	The percentage of individuals living below the poverty line (5 levels)	Poisson regression Age, sex, education, unemployment, single parenthood, dwelling value, dwellings in need of repair	No statistically significant relationship between SES and traffic injuries (RR 1.5; CI 1.1–2.1)
Gagné & Hamel 2009 Canada (Québec province)	Hospitalisations, RTI as motor vehicle occupant, bicyclist & pedestrian	0–14 years R: hospital administrative data system	Area material deprivation for census dissemination areas (quintiles)	Poisson regression Age, sex, residence location, area social deprivation	Children from the least privileged areas have significantly higher RRs than their peers from privileged areas (motor vehicle occupants RR = 1.7; CI 1.3–2.2, pedestrians RR = 3.6; CI 2.7–5.0, bicyclists RR = 1.3; CI 1.1–1.5).
Graham et al. 2005 United Kingdom (England)	Police reported pedestrian casualties	0–16 years R: regional register data	Deprivation index for wards	Negative binomial regression Number of children, volume of traffic flows, physical environment, local road infrastructure	An association between increased deprivation and higher number of pedestrian causalities. For adults (t-statistics 16.0) but stronger association for children (t-statistics 26.4)
Hippsley-Cox et al. 2002 United Kingdom (Trent)	Hospitalisations, RTI as pedestrian, bicyclist and other transport injuries	0–14 years R: regional admissions data	Townsend deprivation index of electoral wards (quintiles)	Poisson regression Rurality, percentage males, ethnicity, distance to nearest hospital	Socioeconomic gradient for RTI among children up to 15 years, especially in those under 5 years that persisted with severity level. The gradient was steepest for pedestrian injuries (adjusted RR = 3.7; CI 2.9–4.5)
Kendrick 1993 United Kingdom (Greater Nottingham)	Police reported pedestrian accidents	0–10 years R: regional register data	Deprivation zones based on aggregated enumeration districts (4 levels)	X^2^-test, Spearman rank correlation coefficients	A significantly higher rate of pedestrian accidents in deprived areas for children 0–4 years (*r*_s_ = 0.61; CI 0.5–0.7) and children 5–11 years (*r*_s_ = 0.68; CI 0.6–0.8)
Lyons et al. 2003 United Kingdom (Wales)	Hospitalisations, pedestrian RTI and nonpedestrian RTI	0–14 years R: routine centralised inpatient register	Townsend deprivation index of electoral tracts (quintiles)	Standardised admission rates, standardised hospitalisation ratios (95% CIs)	Admission rates for pedestrian injuries are substantially higher in more deprived areas (63.2; CI 57.1–69.2) than in the most affluent areas (28.3; CI 23.2–33.3).
Moustaki et al. 2001 Greece (Greater Athens)	Hospitalisations, pedestrian injuries	0–14 years R: emergency department injury surveillance system	% adult household head with higher education degree% of residences with less than one person per room	Chi square, Mantel Haenzel, *t*-test, analysis of variance	Less wealthy towns had an almost twofold excess of pedestrian injuries compared with wealthier ones. The social gradient was steeper outside the residential town (*P* < .001)
Oliver & Kohen 2009 Canada (whole country)	Hospitalisations, RTI as motor vehicle passenger and pedestrian/bicyclist	0–19 years R: hospital morbidity database	Neighbourhood income level based on Dissemination Areas (DA)	Poisson regression, linear trend test Age, sex	In rural areas, children from lower income neighbourhoods have higher hospitalisation rate for injuries as vehicle occupants (hospitalisation rates 5.52; CI 5.1–5.9) than those from the richest neighbourhoods (4.3; CI 3.9–4.7)
Poulos et al. 2007 Australia (New South Wales)	Hospitalisations, RTI as pedestrian, bicyclist, motorcycle rider, motor vehicle occupant	0–14 years R: inpatient register	Index of Relative Socioeconomic Disadvantage of statistical local areas (quintiles)	Negative binomial regression Age, sex	Children in the most disadvantaged quintile are more likely to be hospitalised than children in the least disadvantaged quintile for RTI as pedestrians (IRR = 2.54; CI 1.9–3.4), bicyclists (IRR = 1.30; CI 1.2–1.4), motor vehicle occupants (IRR = 1.84; CI 1.6–2.2), motorcycle rider (IRR = 2.95; CI 2.5–3.5)
Reimers et al. 2008 Sweden (Stockholm county)	Hospitalisations stratified by sex, age and time period (1993–95; 2003–05) motor vehicle rider	10–14 and 15–19 years R: regional inpatient register	Socioeconomic deprivation index of parishes (quintiles)	Poisson regression None	Boys living in areas with the highest level of economic deprivation have lower rates of RTI as motor vehicle rider (10–14 years, RR = 0.26; CI 0.1–0.7, 15–19 years, RR = 0.3; CI 0.2–0.5)
Reimers & Laflamme 2005 Sweden (Stockholm county)	Hospitalisations, RTI as pedestrian, bicyclist, moped rider, car passenger motor vehicle rider	0–15 years R: regional inpatient register	Deprivation index, SES index of parishes (3 levels of each)	Rate ratios None	Higher levels of deprivation negatively influence pedestrian injuries (RR = 1.92; CI 1.2–2.3) and a protective effect on other traffic-related injuries, bicyclists (RR = 0.59; CI 0.5–0.7), moped riders (RR = 0.30; CI 0.2–0.4), car passengers (RR = 0.67; CI 0.3–0.6)
Reimers & Laflamme 2004 Sweden (Stockholm county)	Hospitalisations, RTI as bicyclist, moped rider	10–19 years R: routine centralised inpatient register	Material deprivation, SES, and multi-ethnicity indices for parishes (3 levels of each)	Logistic regression None	Boys in areas with relatively higher concentration of socioeconomic precariousness and immigrant concentration have reduced risk for RTIs as bicyclists (OR=0.4; CI 0.3–0.5) and moped riders (OR=0.6; CI 0.5–0.8)
Silversides et al. 2005 Ireland (North and West Belfast	Injuries seen in emergency department RTI as pedestrian, bicyclist, car passenger	0–12 years R: emergency department register	The Noble economic deprivation index of enumeration districts (2 levels – most versus least deprived areas)	Student's *t*-test None	Children living within the most deprived areas were more likely to be involved in road traffic injuries, pedestrian (RR = 1.32; *P* < .76), bicycle (RR = 2.43; *P* < .22), vehicle (RR = 2.88; *P* < .23)
Turrell & Mathers 2001 Australia (whole country)	Mortality due to motor vehicle traffic accident	0–14, 15–24 years R: Death register	Index of relative socioeconomic disadvantage for statistical local areas, Gini coefficient	Rate ratio with 95% CI	Children in disadvantaged areas have increased mortality due to motor vehicle accidents for males in both age groups (0–14 years, RR = 2.49; *P* < .001, 15–24 years, RR = 2.26; *P* < .001) and for females (0–14 years, RR = 1.4; *P* < .001, 15–24 years, RR = 1.83; *P* < .001)

**Table 4:** Multilevel studies for childhood road traffic injuries: summary of methodological features and results (*n* = 2).

Author & year country (City/region)	Outcome/s B, F, P, D^a^	Age group/s data source^b^	SES measure	Analysis covariates	Results: the level of 95% is used for all confidence intervals (CI)
Kim et al. 2007 South Korea (whole country)	Transportation-related mortality (all types)	0–5 years R: birth and death registers	Father's occupation, mother's education for individual level, deprivation index for districts (5 levels)	Multilevel poisson regression Sex	Deprivation showed a clear positive relationship with mortality by transport-related causes (RR = 1.5—estimated from figure—for 4th quintile compared to first quintile)
Laflamme et al. 2009 Sweden (Stockholm county)	Hospitalisations, RTI as pedestrian, bicyclist, motor vehicle rider	7–16 years R: regional inpatient register	Family disposable income, Townsend deprivation index Congdon index	Multilevel study NLMXED procedure in a two-level model Age	After adjusting for compositional factors, there was still unexplained area variability for injuries among motor vehicle riders

**Table 5:** Individual-level studies for childhood burn, fall, poisoning, and drowning injuries: summary of methodological features and results (*n* = 11).

Author & year country (city/region)	Outcome/s b, F, P, D^a^	Age group/s data source^b^	SES measure	Analysis covariates	Results: the level of 95% is used for all confidence intervals (CI)
Cho et al. 2007 South Korea (whole country)	D Deaths, stratified by sex and age group	10–14 and 15–19 years R: death register, health insurance beneficiary dataset	Parental income (based on insurance contribution - 3 levels)	Cox proportional hazards model None	Drowning deaths showed no socioeconomic gradient among boys or girls for either age group (eg, for boys 10–19 years RR = 1.26, CI 0.82–1.92, p for trend = 0.28, lowest compared to highest income tertile)
Delgado et al. 2002 Peru (Lima)	B Hospitalisations (all burn types)	0–17 years Q: structured questionnaire with guardians	Household income (2 levels), crowding (2 levels), maternal education (2 levels)	Logistic regression (case-control study) No water supply, living room in house, own house, patient is not child of household head	Children in low income (OR=2.8; CI 2.0–3.9) and crowded (OR=2.5; CI 1.7–3.6) households have increased risk of burn injuries compared to those in households with higher income and no crowding; children of mothers with at least a high school education have lower risks compared to those with mothers without this education (OR=0.6; CI 0.5–0.9)
Donroe et al. 2009 Peru (Lima)	P, B, F Severe enough to require medical consultation	0–18 years I: household survey with guardian or with child if aged ≥12 years	Poverty (2 levels), parental education (2 levels)	Logistic regression Sex, age, other SES, overcrowding, number of children in the home	No association between SES and individual injury in multivariate model but increased odds of falls for children who are from homes that are both poor *and *with low parental education (OR=1.30; CI 1.0–1.7). Children in poor households had increased odds of burn injuries (adjusted OR=1.34; CI 1.0–1.8) compared to those in more affluent households
Edwards et al. 2006 United Kingdom (England and Wales)	B Deaths from exposure to smoke, fire, and flames	0–15 years R: population-based death register	Family occupational status (8 levels)	Death rates (95% CI) None	Children from family with the least favourable occupational status had 37.7 (CI 11.6–121.9) times higher death rates than those from the most favourable one
Engström et al. 2002 Sweden (whole country)	F Hospitalisations and deaths combined, stratified by age	0–4, 5–9, 10–14 and 15–19 years R: linkage of health, death and census records	Parental social class (4 levels)	Logistic regression, slope index of inequality, relative index of inequality Parents' country of birth, single parent home, receipt of welfare benefits	No association between SES and risk of fall injuries except among 0–4 year olds (RR = 1.08; CI 1.0–1.1 for children both of unskilled and skilled workers compared with children of intermediate and high level employees)
Forjuoh et al. 1995 Ghana (Ashanti region)	B Injuries with evidence of physical scar	0–5 years I: household survey of caretakers	Maternal education (2 levels)	Logistic regression (case-control study) Presence of pre-existing impairment in child, history of sibling burn, storage of flammable substance in home	Maternal education was not significantly associated with childhood burns (OR=0.76, CI 0.55–1.05 for educated mother compared to a mother without education)
Giashuddin et al. 2009 Bangladesh (randomly selected areas of whole country)	D Deaths and nonfatal injuries separately	1–4 years I: household survey	Assets Index (quintiles)	Concentration index	Drowning morbidity and mortality were 3.8 and 7.0 times higher, respectively, in the least as compared the most deprived quintile. Concentration indices −.21 and −.28, respectively) showed significant inequalities among the groups (*P* < .05)
Gilbride et al. 2006 Canada (Alberta province)	P, B Cases requiring physician consultation	0–17 years R: administrative health database	Receipt of healthcare premium subsidy (as proxy for low SES – 2 levels)	Logistic regression Sex, age	Compared to children from families without subsidies, those from low SES families had higher odds of burns (OR=1.35; CI 1.3–1.4) and poisoning (OR=1.60; CI 1.5–1.7)
Laursen & Nielson 2008 Denmark (whole country)	P, B, F Injuries occurring at home and seen in emergency department. Falls: from ≥1 metre	0–14 years R: national injury register	Parents' education (3 levels), and income (4 levels)	Poisson regression Age, sex, distance from hospital, number of children, age at childbirth, family type, crowding, dwelling type	Increasing injury with decreasing SES for each cause. Compared to children of parents with a tertiary education, those of parents with a primary school education had higher risks of poisoning (RR = 1.9; CI 1.6–2.3), burns (RR = 1.6; CI 1.4–1.9) and high falls (RR = 1.4; CI 1.2–1.7). Compared to children of parents in the most affluent group, those of parents in the lowest income group had higher risks of poisoning (RR = 1.7; CI 1.4–2.1), burns (RR = 1.9; CI 1.6–2.3) and high falls (RR = 1.2; CI 1.0–1.4)
Roberts 1997 United Kingdom (England and Wales)	P, B, F Deaths	0–15 years R: death register	Social class of father (6 levels)	Poisson regression None	Mortality differentials were steepest for fire-related deaths (OR=1.89; CI 1.8–2.0), followed by falls (OR=1.46; CI 1.3–1.6) and poisoning (OR=1.36; CI 1.1–1.6)
Scholer 1998 United States (State of Tennessee)	B House fires resulting in at least one fatality	0–5 years R: linkage of birth certificates, census data & death certificates	Maternal education (4 levels), neighbourhood income (5 levels)	Poisson regression (cohort study) Maternal age, race, marital status, residence, number of children, first prenatal care visit, child sex & gestational age	Low maternal education was positively associated with an increased risk of fatal fire events (RR = 19.36; CI 2.6–142.4 for <12 years education compared to ≥16 years). The association between neighbourhood income and injury did not persist in the multivariate analysis

Note ^a^B=burns, F=falls, P=Poisoning, D=Drowning; ^b^R=register; I= interview, Q=self-administered questionnaire.

**Table 6:** Area-level studies for childhood burn, fall, poisoning, and drowning injuries: summary of methodological features and results (*n* = 17).

Author & year country (city/region)	Outcome/s B, F, P, D^a^	Age group/s data source^b^	SES measure	Analysis covariates	Results: the level of 95% is used for all confidence intervals (CI)
Birken et al. 2006 Canada (urban areas)	FDeaths	0–14 years R: death register	Household income for census tracts (quintiles)	Poisson regression Age, sex	For each unit change in income quintile, from highest to lowest, the risk of death from falls increased by 29% (CI 8%−54%). This did not change over time.
Durkin et al. 1994 United States (Northern Manhattan)	B, F Hospitalisations and deaths combined	0–16 years R: injury surveillance system	Household income (3 levels), education (2 levels), unemployment (2 levels) for census tracts (quartiles)	Regression analysis, rate ratios with 95% CI None	Compared to children living in areas with few low-income households, those in areas with moderate and high numbers of low-income households are more likely to have burn injuries (RR = 1.4; CI 1.1–1.8 and RR = 1.6; CI 1.3–2.1, respectively) and fall injuries (RR = 1.5; CI1.3–1.8 and RR = 1.9; CI 1.5–2.2, resp.)
Edwards et al. 2008 United Kingdom (England)	F Serious hospitalised injuries	0–15 years R: centralised inpatient registers	Index of Multiple Deprivation (deciles)	Negative binomial regressionEthnicity, % households with no car, % lone-parent families	The increased risk of falls with greater deprivation disappeared after adjustment (OR=0.57, CI 0.24–1.33 for most deprived decile compared to least deprived one)
Faelker et al. 2000Canada (Kingston)	F Injuries seen in emergency departments	0–19 years R: population-based injury surveillance system	% people living below poverty line for enumeration areas (5 levels)	Poisson regression Age, sex, other SES variables	Gradient of increasing injury with decreasing income; RR = 1.42 (CI 1.21–1.68) for children in poorest quintile compared to those in richest quintile
Gagné & Hamel 2009 Canada (Québec province)	P, B, F All, and severe, hospitalised injuries; 6 subdiagnoses of falls	0–14 years R: hospital administrative data system	Area material deprivation for census dissemination areas (quintiles)	Poisson regression Age, sex, residence location, area social deprivation	Hospitalizations were associated with deprivation, especially for severe injuries. Compared with children in the least deprived quintile, those in the most deprived quintile had higher hospitalisation rates for fire and burn (RR = 2.05; CI 1.5–2.7), and poisoning (RR = 1.68; CI 1.4–2.0) injuries. Associations only significant for particular types of falls
Groom et al. 2006 United Kingdom (East Midlands)	P Hospitalisations, 2 broad and 7 narrow subdiagnoses	0–4 years R: hospital records	Townsend deprivation index of electoral wards (quintiles)	Negative binomial regressionPercentage males, ethnicity, rurality, distance from nearest hospital	Unintentional poisoning was higher among children in the most deprived wards than those in the least deprived. For all poisonings combined, RR = 2.28 (CI 1.78–2.91) for children in poorest quintile compared to those in richest quintile. Gradients were particularly steep for benzodiazepines, antidepressants, cough and cold remedies, and organic solvents
Hippisley-Cox et al. 2002 United Kingdom (Trent)	P, B, F Hospitalisations	0–14 years R: regional admissions data	Townsend deprivation index of electoral wards (quintiles)	Poisson regressionPercentage males, ethnicity, rurality, distance from nearest hospital	Gradient of increasing injury admissions with increasing deprivation. Compared with children in the least deprived quintile, those in the most deprived quintile had a higher admission rate for poisoning (RR = 2.98; CI 2.7–3.3), burns and scalds (RR = 3.49; CI 2.8–4.3), and falls (RR = 1.53; CI 1.5–1.6)
Istre et al. 2002 United States (Dallas City)	B Residential fire-related injuries resulting in emergency medical treatment, hospitalisation or death	0–19 years R: linkage of emergency medical services, hospital, medical examiner, and fire department records	Census tract median income (5 levels)	Chi squared for trend None	There was a marked gradient in the rate of fire-related injuries by income of census tracts. Injury rate in lowest income census tract group was 7.0, compared with 3.1, 1.2, 0, 0 for each successively higher median income grouping (*P* < .01 by *χ* ^2^ for trend)
Laflamme & Reimers 2006 Sweden (Stockholm County)	F Hospitalisations; 7 subdiagnoses; 2 severity levels	0–5 and 6–15 years R: routine centralised inpatient registers	Socioeconomic circumstances index and SES index of parishes (3 levels of each)	Logistic regression None	Results varied by age, fall injury type and severity. Deprived socioeconomic circumstances and low SES typically associated with reduced risk, especially for 0–5 year olds (eg, for falls on the same level, OR=0.63, CI 0.5–0.7 for children living in poor as compared to high socioeconomic circumstances)
Lyons et al. 2003 United Kingdom (Wales)	P, B, FHospitalisations; burns including scalds	0–14 years R: routine centralised inpatient register	Townsend deprivation index of electoral tract (quintiles)	Standardised admission rates, standardised hospitalisation ratios (95% CIs)	Admission rates are significantly higher in more deprived quintiles for each cause. For poisoning, burns, and falls, respectively, rates in the most deprived quintile were 663.6 (CI 622.7–704.5), 81.1 (CI 66.6–95.6), and 1384.0 (CI 1326.3–1441.6) compared to rates in the least deprived quintiles 341.3 (CI 299.3–383.4), 34.9 (CI 21.2–48.6), and 953.9 (CI 889.3–1018.4)
Poulos et al. 2007 Australia (New South Wales)	P, B, FHospitalisations; 2 subdiagnoses of falls	0–14 years R: inpatient register	Index of Relative Socioeconomic Disadvantage of statistical local areas (quintiles)	Negative binomial regression Age, sex	Children in the most disadvantaged quintile were more likely than the least disadvantaged quintile to be hospitalized for poisoning (IRR = 1.52; CI 1.4–1.7) and fire and burn (IRR = 1.95; CI 1.7–2.3) injuries. Children in the most disadvantaged quintile at reduced risk of falls (IRR = 0.78; CI 0.7–0.8)
Reimers et al. 2008 Sweden (Stockholm county)	F Hospitalisations, stratified by sex, age and time period (1993–95; 2003–05)	10–14 and 15–19 years R: regional inpatient register	Socioeconomic deprivation index of parishes (quintiles)	Poisson regression None	For boys, greater deprivation was associated with increased risk of injury only in the first time period and only for the most deprived (ages 10–14years RR = 1.62; CI 1.0–2.6) and intermediately deprived (ages 15–19 years RR = 1.69; CI 1.0–2.8) quintiles. Significant results were present only for girls aged 15–19 years—in the first time period, there was a protective effect of deprivation (RR = 0.65; CI 0.4–1.0 for most deprived), in the second time period, an aggravating effect (RR = 2.62; CI 1.3–5.5 for most deprived)
Reimers & Laflamme 2005 Sweden (Stockholm county)	P, B, F Hospitalisations	0–15 years R: regional inpatient register	Deprivation index, SES index of parishes (3 levels of each)	Rate ratios None	Compared to high SES areas, areas with a greater concentration of people with low SES increased the risk of burn (RR = 2.30; CI 1.5–3.4) and poisoning (RR = 1.65; CI 1.2–2.3) but did not impact on the risk of fall injuries. Moderate, compared to low, deprivation was associated with reduced risk of burn injuries (RR = 0.36; CI 0.2–0.6)
Reimers & Laflamme 2004 Sweden (Stockholm county)	F Hospitalisations, 4 subdiagnoses, stratified by sex	10–19 years R: routine centralised inpatient register	Material deprivation, SES, and multi-ethnicity indices for parishes (3 levels of each)	Logistic regression None	Results varied by sex, fall injury type and index, associations were both aggravating and protective (eg, for falls on the same level OR= 1.22; CI 1.1–1.4 for high, as compared to low, deprivation for boys; but OR= 0.82; CI 0.7–1.0 for girls)
Shai & Lupinacci 2003 United States (Philadelphia)	B Deaths from residential fires	0–14 years R: fire department data	Education level and household income of census tracts (2 levels each)	Logistic regression % children aged under 15; age of house, single-parent households	Low-income tracts had higher odds of experiencing at least one fatal fire-related death (OR=3.18; CI 1.6–6.5)
Silversides et al. 2005 Ireland (North and West Belfast)	P, B, F Injuries seen in emergency department, 2 subdiagnoses of falls, burns including scalds	0–12 years R: emergency department register	The Noble economic deprivation index of enumeration districts (2 levels - most vs. least deprived areas)	Student's *t*-test None	Although burn, fall and poisoning injuries were considerably higher in the most, as compared to the least, deprived areas, the difference in rates only reached significance for falls <1 metre (RR = 1.90; *P* < .02)
Van Niekerk et al. 2006 South Africa (Cape Town)	B Hospitalisations	0–12 years R: hospital records	Housing conditions, socioeconomic barriers, and child dependency indices for residential areas (3 levels of each)	Logistic regression None	Children living in residential areas with poor (OR=2.39; CI 2.1–2.8) or impoverished (OR=3.33; CI 2.8–3.9) housing conditions; with medium (OR=1.94; CI 1.6–2.3) or severe (OR=3.61; CI 3.0–4.3) socioeconomic conditions; and with high (OR=1.80; CI 1.4–2.3) child dependency had greater odds of burn injuries than those living in areas with the most favourable levels of these dimensions

Note ^a^B=burns, F=falls, P=Poisoning, D=Drowning; ^b^R=register; I= interview, Q=self-administered questionnaire.

**Table 7:** Multilevel studies for childhood burn, fall, poisoning, and drowning injuries: summary of methodological features and results (*n* = 1).

Author & year country (city/region)	Outcome/sB, F, P, D^a^	Age group/s data source^b^	SES measure	Analysis covariates	Results: the level of 95% is used for all confidence intervals (CI)
Kim et al. 2007 South Korea (whole country)	D, F Deaths	0–5 years R: birth and death registers	Father's occupation, mother's education for individual level, deprivation index for districts (5 levels)	Multilevel poisson regression Sex	Deprivation showed a clear positive relationship with mortality by drowning (RR = 1.7—estimated from figure—for 4th quintile compared to first quintile), but not by falls, after controlling for individual-level variables

Note ^a^B=burns, F=falls, P=Poisoning, D=Drowning; ^b^R=register; I= interview, Q=self-administered questionnaire.
